# Clinical Aspects of Uncomplicated and Severe Malaria

**DOI:** 10.4084/MJHID.2012.026

**Published:** 2012-05-04

**Authors:** Alessandro Bartoloni, Lorenzo Zammarchi

**Affiliations:** Infectious Diseases Unit. Department of Critical Care Medicine and Surgery, University of Florence, Florence, Italy

## Abstract

The first symptoms of malaria, common to all the different malaria species, are nonspecific and mimic a flu-like syndrome. Although fever represents the cardinal feature, clinical findings in malaria are extremely diverse and may range in severity from mild headache to serious complications leading to death, particularly in falciparum malaria. As the progression to these complications can be rapid, any malaria patient must be assessed and treated rapidly, and frequent observations are needed to look for early signs of systemic complications.

In fact, severe malaria is a life threatening but treatable disease. The protean and nonspecific clinical findings occurring in malaria (fever, malaise, headache, myalgias, jaundice and sometimes gastrointestinal symptoms of nausea, vomiting and diarrhoea) may lead physicians who see malaria infrequently to a wrong diagnosis, such as influenza (particularly during the seasonal epidemic flu), dengue, gastroenteritis, typhoid fever, viral hepatitis, encephalitis. Physicians should be aware that malaria is not a clinical diagnosis but must be diagnosed, or excluded, by performing microscopic examination of blood films. Prompt diagnosis and appropriate treatment are then crucial to prevent morbidity and fatal outcomes. Although *Plasmodium falciparum* malaria is the major cause of severe malaria and death, increasing evidence has recently emerged that *Plasmodium vivax* and *Plasmodium knowlesi* can also be severe and even fatal.

## Introduction

Malaria is usually transmitted by the bite of an infected female anopheline mosquito, although blood-borne transmission (blood or blood products transfusion, transplantation, needle-sharing among intravenous drug addicts, accidental nosocomial transmission) or congenital transmission may occur.^1^

In the case of mosquito-borne transmission, sporozoites, the infective plasmodial stage injected along with saliva into subcutaneous capillaries, disappear from the blood within approximately 45 minutes of the bite and enter liver parenchimal cells (hepatocytes). Inside the hepatocytes, each sporozoite begins a phase of asexual reproduction resulting in the formation of a schizont, which contains thousands of merozoites. The rupture of mature schizonts generates the liberation of merozoites into the bloodstream. The hepatic phase of parasite development (hepatic schizogony) lasts on average between 5.5 (*Plasmodium falciparum*) and 15 days (*Plasmodium malariae*). In case of *Plasmodium vivax* and *Plasmodium ovale* infections, a proportion of parasites may remain dormant in hepatocytes as hypnozoites for several months up to 5 years. From the clinical point of view, the hepatic schizogony is asymptomatic, as only a few number of liver cells is infected.^2^

Once in the bloodstream, merozoites reach and invade red cells rapidly to start a process of asexual multiplication (erythrocytic schizogony). Within the red blood cell, merozoites mature to trophozoites and schizonts, which then rupture liberating the new generation of merozoites to invade other red blood cells and thus continue the erythrocytic cycle.

At the time of schizont rupture, the release of malaria parasites and erythrocytic material into the circulation induce the pathophysiology process of malaria and the onset of symptoms.

The activation of the cytokine cascade is responsible of many of the symptoms and signs of malaria.

## Uncomplicated Malaria

While the interval from time of infection until parasites become detectable in the blood is termed prepatent period, with the term of incubation period is defined the interval between infection and the onset of symptoms. The duration of incubation period is influenced by several factors such as the species of infecting parasites, the way of parasite transmission, the degree of previous immune status of the host, the chemoprophylactic use of antimalarial drugs, and probably the density of parasite inocula. Incubation period ranges from 9 to 30 days with *P. falciparum* infections, tending to present the shortest, and *P. malariae* the more prolonged times. In most of *P. falciparum* and *P. vivax* malaria, the incubation period is approximately two weeks. In blood-induced infections, the incubation period is usually shorter with symptoms developing within 10 days of transfusion for *P. falciparum*, 16 days for *P. vivax*, and 40 days or longer for *P. malariae*.^1^ As far as the degree of previous protection possessed by the infected subject is concerned, it is known that effective immunity prolongs incubation period and reduces level of parasitemia and clinical manifestations. Low asymptomatic parasitemia may persist in migrants from endemic areas long after their arrival in the host country,^1^ and delayed clinical presentation of *P. falciparum* have been described as long as 2,^3^ 4^4^ or even 8 years^5^ after subjects have left malaria-endemic areas. Pregnancy and co-infection with HIV have been associated with late presentation of malaria caused by *P. falciparum* in immigrants.^6^

Prolonged incubation period may also be caused by the use of antimalarial drugs that, although ineffective, may impact on the parasite multiplication rate.

The clinical manifestations of malaria are dependent on the previous immune status of the host.^7^ In areas where endemicity of *P. falciparum* malaria is stable, severe malaria most commonly occurs in children up to 5 years of age, while is less common in older children and adults because of the acquisition of partial immunity. In areas of lower endemicity, the age distribution of severe malaria is less well defined and may also occur in adult semi-immune persons.

The first symptoms of malaria, common to all the different malaria species, are nonspecific and mimic a flu-like syndrome. The hallmark of malaria is fever. Up to two days before the onset of fever, prodromal symptoms, such as malaise, anorexia, lassitude, dizziness, with a desire to stretch limbs and yawn, headache, backache in the lumbar and sacroiliac region, myalgias, nausea, vomiting and a sense of chillness may be experienced.^8^ The fever is usually irregular at first and the temperature rises with shivering and mild chills. After some days fever tends to become periodic depending on the synchronized schizogony. In fact, for unknown reasons development of asexual blood stage parasites becomes synchronous after some days with a periodicity depending on the length of the asexual cycle.

### Plasmodium vivax *and* Plasmodium ovale

In *P. vivax* and *P. ovale* infection, if left untreated, asexual cycles become synchronous after 5 to 7 days causing periodic febrile paroxysms. The classical malaria paroxysm presents three stages: a cold stage, followed by a hot stage with a terminal sweating stage. The cold stage is typically characterized by a sudden onset with a feeling of extreme coldness. The subject may shiver and his or her teeth may chatter. In virtue of an intense peripheral vasoconstriction phenomenon, the skin is cold, dry, pale, cyanosed and sometimes goose-pimpled.^1^,^7^,^8^ The subject feels the need to cover himself with all available blankets he can reach, but without obtaining comfort. During this initial stage, that usually lasts 10–30 minutes and only occasionally up to 90 minutes, the temperature rises gradually to a peak (usually between 39° and 41° C). Eventually, the shivering ceases and the second or hot stage starts. The skin is now hot and dry and the face flushed, with the subject that feels heat and discards blanket. The temperature may further rise and reach hyperpyrexial levels. Vomiting is common in this phase and sometimes diarrhoea, severe retroorbital headache, parched throat, extreme thirst, and altered consciousness are also present. In young children convulsion may occur. Within 2–6 hours, the subject enters the third stage of the paroxysm, the sweating stage, with sudden profuse sweating, appearing first at the temples, and rapidly becoming generalized and copious. The temperature falls rapidly and the subject feels well, although extremely tired, and usually falls asleep. The sweating stage lasts 2 to 3 hours, with the entire paroxysm, which more frequently begins in late afternoon or evening, lasting 6–10 hours.

At the physical examination, splenomegaly may be present during the acute attack but is more commonly observed after the second week of the attack. Rupture of an enlarged spleen is a possible, although rare, complication. The liver may also be enlarged and palpable.

As far as laboratory findings are concerned, some degree of anaemia and reticulocytosis may be present due to lisys of parasitized and unparasitized red blood cells. Thrombocytopenia is common and sometimes mild leucopenia is present. By examining blood films, representatives of all developmental forms of the asexual parasite, from the early ring to mature schizont, may be observed, while gametocytes are usually present after a period of about a week. The density of parasitemia seldom exceeds 2% of the erythrocytes.

In *P. vivax* and *P. ovale* infection, schizogony occurs every 48 hours so that the febrile paroxysm occurs every third day (tertian fever). During the intervals between febrile paroxysm, the subject is usually afebrile and feels well. If left untreated, the attacks may last from a few weeks to some months and then spontaneously and gradually resolve. In *P. vivax* and *P. ovale* infection, after a quiescent or latent period, relapse may occur with renewal of clinical symptoms and asexual parasitemia. This phenomenon is due to reinvasion of the blood by merozoites produced when hypnozoites awake from dormancy and develop into hepatic schizont. Relapses occur weeks, months, or even years after the primary infection with different timing related to the *P. vivax* strain, geographical origin of the infection and the previous use of inadequate antimalarial treatment. From the clinical point of view, relapse is similar to the first attack, except for a more abrupt onset and the absence of the initial period of irregular fever, as the infection is more synchronous. The relapse is usually less severe and of shorter duration than the first attack.

Infections with *P. vivax* and *P. ovale* are rarely complicated. However, there is increasing evidence of serious and fatal complications due to *P. vivax* infection.^9^–^12^

### Plasmodium falciparum

In *P. falciparum* malaria, the onset of fever occurs after few days of prodromal symptoms started during the last days of the incubation period (normal range 9–14 days). At first, fever is irregular, but usually occurs daily. It may be intermittent or continuous, and shows no sign of periodicity until the illness has continued for a week or more. The symptoms present in the prodromal phase continue and increase, so configuring a flu-like syndrome. Anorexia, dispepsia, epigastric discomfort, nausea, vomiting and watery diarrhoea are frequent and may be misdiagnosed as a gastrointestinal infection. Herpes labialis may be present. A dry cough and an increase in the respiration rate may be observed, arising the suspect of an acute respiratory infection. Other non-specific physical findings are tender hepatosplenomegaly, orthostatic hypotension, and some degree of jaundice. The pulse may be rapid (100 to 120 beats/min) and the blood pressure may be low (90–100 mmHg, systolic).^13^ When periodic febrile paroxysms occur, they may be daily (quotidian), every third day (tertian) or at about 36-hour intervals (subtertian).

Routine laboratory exams may show little or no anaemia in mild cases and in otherwise healthy individuals. When present, it is usually normochromic and normocytic. The reticulocyte count is normal or depressed despite haemolysis, and increases only after the parasitemia is cleared. Serum haptoglobin may be undetectable indicating haemolysis. However, the degree of anaemia may greatly vary and is not always an indication of the severity of the attack.

The white cell count is usually normal, but leucopenia with a count of 3,000–6000 cells/μl may be observed. Thrombocytopenia is common (usually around 100,000/μl) and does not correlate with severity. Serum transaminases and lactic dehydrogenase usually are moderately elevated as well as bilirubin concentrations, both conjugated (hepatocyte disfunction and/or cholestasis) and unconjugated (haemolysis). Prothrombin and partial tromboplastin time may be prolonged while fibrinogen levels are usually elevated. C-reactive protein and procalcitonin value are usually increased in both uncomplicated and severe malaria with levels correlated with parasitemia and prognosis according with some studies.^14^–^16^ Hyponatremia is not uncommon and, in some cases, a transient increase in serum creatinine and blood urea nitrogen may be observed. Urine analysis reveals albuminuria and urobilinogen.

Examination of peripheral blood film mostly reveals the presence of early trophozoites (typical small ring forms), often numerous, with multiple infected red blood cells. Late trophozoites, as well as schizonts, are seldom observed, being their presence an indicator of very heavy infections and poor prognosis. Gametocytaemia usually occurs in the second week after the onset of parasitemia and may persist for some weeks after cure.^1^

Although the subject may not appear very ill, serious complications may develop at any stage. In non-immune people *P. falciparum* malaria may progress very rapidly to severe malaria unless appropriate treatment is started. If the acute attack is rapidly diagnosed and adequately treated, the prognosis of falciparum malaria is good, even if complications may still occur. The response to treatment is usually rapid with resolution of fever and most symptoms within 3 days.^7^ Recrudescence with renewal of clinical manifestation and/or parasitemia, due to persistent erythrocytic forms, may occur.

### Plasmodium malariae

*P. malariae* causes the mildest and most persistent form of malaria infection.^1^ After an incubation period that is never less than 18 days, but that may be up to 30–40 days, prodromal symptoms resembling those of vivax malaria proceeds the onset of fever. The clinical picture of the primary attack is similar to that of vivax malaria. The onset is often insidious, but febrile paroxysms, often occurring in the late afternoon, show well synchronized schizogony from an early stage and are typically separated by intervals of 72 hours (quartan malaria).

As far as laboratory findings are concerned, anaemia is less pronounced and leucopenia may be observed. Parasitemia rarely exceeds 1% of erythrocytes. Representatives of all developmental forms of the asexual parasite are usually present. Left untreated, the acute attack is self limiting but may last for several months before spontaneous remission occurs.^1^ Severe complications of *P. malariae* infection are rarely observed. However, recrudescences may occur, more frequently during the first year and then at longer intervals, even after 30–50 years. *P. malariae* has no hypnozoite form, so recrudescences arise from persisting blood stage. Asymptomatic *P. malariae* parasitemia in blood donors may cause transfusion malaria.

*P. malariae* infection is associated with development of a nephrotic syndrome. First clinical manifestation often onsets before 15 years of age. Classically, edema and ascites are present and accompanied by laboratory evidence of heavy proteinuria, hypoalbuminemia and hyperlipidemia. *P. malariae* parasitemia is common in children, but not in adults. Transient clinical remissions with period of asymptomatic proteinuria are frequent but progressive deterioration and development of renal failure often occur within 3 to 5 years.^13^

### Plasmodium knowlesi

*P. knowlesi* is the species of plasmodium most recently identified as agent of human malaria.^17^ Until few years ago, it was known as agent of chronic infection of the long-tailed (*Macaca fascicularis*) and pig-tailed (*Macaca nemestrina*) macaques.^18^ *P. knowlesi* malaria is the most common locally acquired human malaria in Malaysian Borneo (∼70% of malaria cases),^19^ with the disease also reported from other countries of southern and eastern Asia.^20^ On the basis of clinical features, it is not possible to distinguish knowlesi malaria from vivax or falciparum malaria.^19^ The development of hyperparasitemia and other complications are fairly common.^19^ Concerning diagnosis, the identification of *P. knowlesi* infection by using microscopy only is difficult because it is very similar to *P. malariae.*^21^ Polymerase Chain Reaction (PCR) is currently the method of choice to obtain a certain diagnosis.^17^

## Severe Malaria

Most of the severe malaria complications occur in non immune subjects with falciparum malaria and involve central nervous system (cerebral malaria), pulmonary system (respiratory failure), renal system (acute renal failure) and/or hematopietic system (severe anaemia). As the progression to these complications can be rapid and severe malaria is a potentially fatal disease, any malaria patient must be assessed and treated rapidly, and frequent observations are needed to look for early signs of systemic complications.^1^

According to the most recent definition by World Health Organization (WHO), in a patient with *P. falciparum* asexual parasitaemia and no other obvious cause of symptoms, the presence of one or more of the clinical or laboratory features reported in **Table 1**, classifies the patient as suffering from severe malaria.^22^ This definition has been proposed to assist clinical and epidemiological descriptions. In clinical practice, any subject for whom severe malaria is suspected, must be treated as having severe malaria, although not clearly fulfilling the proposed WHO criteria.^7^

### Cerebral malaria

In order to allow comparability of clinical and therapeutic findings, a strict definition of cerebral malaria was recommended.^23^ In accordance to the most recent WHO definition cerebral malaria is a severe *P. falciparum* malaria with cerebral manifestations, usually including coma (Glasgow coma scale < 11, Blantyre coma scale < 3). Malaria with coma persisting for > 30 minutes after a seizure is considered to be cerebral malaria.^22^

However, in clinical practice, subjects with any degree of altered consciousness and any other sign of cerebral dysfunction should be treated for severe malaria. Clinical evolution and neurologic findings are highly variable. Coma may onset suddenly or gradually, with initial drowsiness (always worrying symptom), confusion, disorientation, delirium or agitation.^1^,^7^,^8^,^23^ Subjects with cerebral malaria may be open-eyed but non-seeing, and present disconjugate gaze and nistagmus. Sustained ocular deviation, generally upward or lateral, may be observed. They may exhibit various forms of abnormal posturing, including decerebrate rigidity (extensor posturing, with arms and legs extended), decorticate rigidity (extensor posturing, with arm flexed and legs extended), and opisthotonus. Neck rigidity and photophobia are rare symptoms, but some resistance to passive neck flexion, in absence of other signs of meningeal irritation, is not uncommon. Seizures, generalized or sometimes focal, are common, particularly in children. Electroencephalographic abnormalities are non-specific. When lumbar puncture is performed in order to differentiate cerebral malaria from other causes of encephalopathy, the cerebral spinal fluid (CSF) is clear and shows a mild lymphocyte pleocytosis (rarely more than 10 cells/μl), and increased protein concentrations (rarely exceeding 150 mg/dL). The blood/CSF glucose ratio is normal, while the lactate concentration of lactate is raised. Examination of fundus may evidence retinal haemorrhages (6–37% of cases), while papilloedema is rarely seen in cerebral malaria.^24^–^27^

If left untreated, cerebral malaria is probably nearly always fatal.^7^ Even when treated, cerebral malaria has an approximate 20% of mortality rate in adults and 15% in children.^7^ Among subjects who survive, the recovery is relatively rapid with complete reversibility of neurological signs and symptoms. However, neurological sequelae may occur. The most common manifestations are psychosis, cranial nerve lesions, extrapyramidal tremor, ataxia, polyneuropathy and seizures. Most of these neurologic disturbances are transient, resolving few days to several weeks after their onset. In some cases, particularly in children, residual neurological deficit may occur after severe malaria. Language disorder, motor deficits, cognitive impairments and epilepsy have been reported in childhood following recovery from cerebral malaria.^28^–^36^

### Respiratory failure

Respiratory failure may rapidly develop at any stage of an acute malaria but, in many cases, occurs when subjects are already recovering, parasitemia is cleared, and in comatose subjects, consciousness has been regained.^1^ Pregnant women are particularly prone to develop acute respiratory failure. The clinical picture is indistinguishable from the adult respiratory distress syndrome that develops as a result of a systemic or pulmonary infection, severe trauma or other systemic or pulmonary insults. Tachypnea, dispnea, and scattered rales and ronchi on auscultation are the earliest warnings. Patients with severe hypoxemia may require mechanical ventilation.

### Acute renal failure

While biochemical evidence of renal dysfunction is frequently observed in otherwise uncomplicated malaria, acute renal failure is another complication of falciparum malaria, much more common in adults than among children. Malaria acute renal failure may be defined as a urine output of <400 mL in 24 h in adults, or 12 mL/kg in 24 h in children, in spite of rehydration, and a serum creatinine of more than 265 μmol/l (>3.0 mg/dL). In practice for initial assessment, the serum creatinine alone is used.^22^ Acute renal failure may be present at the subject admission in a context of a multiple organ involvement, or occurring in the recovery phase of severe malaria. In the former case prognosis is worse. Oliguria is the main feature of acute renal failure, although urine output may also be normal or increased. In oliguric patients, levels of blood urea nitrogen and serum creatinine are elevated and hyperkalaemia, hyperphosphatemia, hypocaelcemia, and metabolic acidosis usually develop. Dialysis may be necessary in some cases.

### Jaundice and hepatic dysfunction

Jaundice is common in adult subjects with severe malaria, while relatively rare in children. It is generally mild or moderate, but in some cases may be marked. Among the jaundiced subjects, hyperbilirubinemia may be predominantly unconjugated, in case of extensive haemolysis of both parasitized and unparasitized erythrocytes, or conjugated, indicating hepatocytic disfunction. Apart from jaundice, signs of severe liver cell damage are uncommon. Concentrations of transaminases may be increased up to ten fold, but never to the level observed in viral hepatitis. The liver, as well as the spleen, is enlarged and tender, especially in young children and non immune adults.

### Severe anaemia

Severe malaria is associated with development of anaemia, usually normochromic and normocytic. However, in endemic areas the morphology may be influenced by the nutritional status of the subject and some helminthiasis, resulting in an associated microcytic (iron deficiency) and macrocytic (folic acid deficiency) component. The causative mechanisms are multifactorial, including haemolysis of infected and uninfected red blood cells, inappropriate bone marrow response, and numerous other individual factors (e.g. bacteraemia, HIV, hookworm, G6PD deficiency, vitamin A and vitamin B12 deficiency).^37^ In areas of high endemicity, severe anaemia is a particular problem in children, where it can be fatal.^38^ In epidemic rather than endemic areas, severe anaemia may also develop in adult population and pregnant women, especially primiparae, are those at major risk. In accordance with the WHO definition, severe malarial anaemia is defined in the presence of a haemoglobin level of < 5 g/dL, or haematocrit < 15%.^22^ The UK malaria treatment guidelines define severe anaemia when haemoglobin level is <8 g/dL.^39^ Pathological consequences of anaemia are particularly likely with such degree of anaemia, however they are also importantly related to the rapidity at which it develops. In this perspective, decisions regarding blood transfusion should be taken at individual level. Children with severe anaemia and respiratory distress will benefit from transfusions that may be lifesaving.^13^

### Hypoglicemia

Hypoglicemia has been detected in approximately 8% of adults and up to 30% of children with cerebral malaria.^7^ Hypoglicemia is a particular complication in pregnancy. It may be present before starting antimalarial treatment, representing a sign of poor prognosis. Hypoglicemia can also result from rapid infusions or prolonged treatment with quinine, due to its capability to stimulate insulin secretion. Early diagnosis is essential in order to assure a prompt treatment that may be lifesaving, but symptoms may be easily overlooked. In fact, classical symptoms of hypoglicemia, such as those induced by adrenaline secretion (sweating, tachycardia, breathlessness, tremor, anxiety and hunger) or those due to dysfunction of central nervous system (impairment of consciousness, seizures, extensor posturing), are difficult to distinguish from those caused by malaria. Any subject with malaria and altered behaviour or consciousness, or seizure, should be evaluated immediately for blood glucose level and, if this cannot be possible, assumed as hypoglicemic and given glucose.

### Blackwater fever

Blackwater fever is a syndrome presenting with passage of mahogany-coloured (or ‘Coca-Cola’-coloured) urine in association with severe intravascular haemolysis. The dark urine is due to haemoglobinuria.^40^ Blackwater fever occurs in subjects with G-6PD deficiency who take antioxidant drugs or foodstuffs, in subjects with G-6PD deficiency who have acute malaria after the administration of quinine or artemisinin derivative, but also in subject with acute, often severe malaria, who have normal red cell G-6PD concentrations.^41^ Blackwater fever has usually a good prognosis resolving without complications. Only a minority of subjects may develop acute renal failure as a consequence of acute tubular necrosis.^42^,^43^

### Complicating and associated infections

Bacterial infection may arise in subjects with severe and uncomplicated malaria. Health care acquired infections such those associated with use of device (i.e. indwelling catheter or vascular line) or with the poor general condition of the patient (i.e. aspiration pneumonia or infected decubitus ulcer in patients with cerebral malaria and prolonged coma) are possible.^41^ Associated bacteraemia, mainly caused by gram negative bacteria, may coexist in severe and uncomplicated malaria and should be suspected, especially in subjects with hypotension and unexplained deterioration despite parasitemia clearance.^22^,^41^ The threshold for administering antibiotic treatment should be low in severe malaria.^22^ A recent study carried out in a cohort of 506 Kenyan children with malaria found 11,7% prevalence of bacteraemia, with non typhoidal *Salmonella* spp being the most common isolates (42,4%).^44^ Several mechanisms are invoked to explain the susceptibility to bacteraemia during malaria. Haemolysis can increase the susceptibility to invasive salmonellosis,^45^ accumulation of haemozoin pigment in monocytes impairs diverse macrophage functions,^46^ and severe malaria leads to bacterial seeding of the bloodstream because of microvascular parasite sequestration in gut mucosa.^47^

### Non-falciparum severe malaria

Although *P. falciparum* malaria is the major cause of severe malaria and death, increasing evidence has recently emerged that *P. vivax* can also be severe and even fatal.^48^–^51^ Besides the rare fatal haemorrhage due to the traumatic or spontaneous rupture of an enlarged spleen, other complications of *P. vivax* infection have been reported, including complications of seizure, shock, jaundice, renal failure, severe anaemia,^51^–^54^ coma^48^,^49^ and acute respiratory distress syndrome.^55^ Recent studies conducted in Indonesia and Papua New Guinea showed rates of severe malaria in *P. vivax* infections as high or higher than those observed in *P. falciparum* malaria.^48^,^49^ *P. ovale* malaria is rarely severe. However, respiratory distress has also been reported as a rare complication of ovale malaria.^56^,^57^ As far as *P. knowlesi* malaria is concerned, most cases are uncomplicated but complications are possible, and in a study conducted in Malaysia, approximately 1 in 10 patients developed potentially fatal complications, with a case fatality rate of 1.8%.^19^ The most frequent complication was respiratory distress, but also multiorgan failure, hypoglicemia, and lactic acidosis were observed.

## Returning Travelers

Imported malaria is an increasing public health problem in many non endemic countries. Although some cases occur in migrants and refugees, returning travelers from tropical and subtropical areas represent the largest group of persons given a diagnosis of malaria.

The high fatality rate is disturbing, ranging from 0.4% to 3% in Europe.^58^,^59^ *P. falciparum* is responsible for nearly all of these deaths. The fatal outcome in malaria cases is attributable to different contributing factors, including failure to take or adhere to recommended prophylaxis, to promptly seek medical care in presence of symptoms, to promptly diagnose and initiate appropriate antimalarial treatment. The protean and nonspecific clinical findings occurring in malaria (fever, malaise, headache, myalgias, jaundice and sometimes gastrointestinal symptoms of nausea, vomiting and diarrhoea) may lead physicians who see malaria infrequently to a wrong diagnosis, such as influenza (particularly during the seasonal epidemic flu), dengue, gastroenteritis, typhoid fever, viral hepatitis, encephalitis. It is not uncommon that the correct diagnosis is performed at the emergency admission of a subject with fever and signs of cerebral involvement or other complications, having being visited a few days before by a physician who had prescribed antipyretic and antibiotic treatment.

## Specific Population at Increased Risk of Developing Severe Malaria

Non immune pregnants in second and third trimester are at increased risk of developing life threatening malaria and are particularly susceptible to develop pulmonary oedema and hypoglycemia. Foetal death and premature labour are common.^60^–^63^ People with immunosuppression related to HIV show an impaired immune control of malaria. There is an increasing risk of illness, increased parasitemia and severe malaria. Therapeutic responses to antimalarial treatment are impaired so treatment failure rates are increased. Drug interactions between antiretrovirals and antimalarials have not yet been studied adequately. Trimethoprim-sulphamethoxazole prescribed as prophylaxis for opportunistic infection gives some protection also for malaria. Co-infected pregnant women are at risk for higher parasite density, anaemia and malarial infection of the placenta. Children born to women with HIV and malaria infection have low birth weight and are more likely to die during infancy.^64^–^69^ Malaria in non-HIV immunocompromised subjects is less studied. Malaria in transplant recipients can be caused by graft-born or blood born infection^70^,^71^ or reactivation of previous infection due to immunosuppression.^72^ The clinical picture of malaria in transplant recipients is usually severe, owing to the impaired immune response.^73^ It is characterized by pyrexia, which may lack the typical periodicity or rigors. Anaemia is severe, being typically haemolytic and occasionally haemophagocytic.^74^ Acute graft dysfunction may occur as a consequence of the haemodynamic consequences of falciparum infection.^75^ Presence of the sickle cell trait confers some protection against malaria, however, for those with homozygous sickle-cell disease, malaria is regarded as a significant cause of morbidity and mortality, producing further haemolysis against the background of that due to sickle-cell disease itself.^76^ Subjects who have no spleen or whose splenic function is severely impaired are at particular risk of severe malaria, and malarial parasitaemia in asplenic individuals may rise rapidly to very high levels.^77^ Post-splenectomy episode of *P. falciparum* malaria has been reported in immigrants.^78^

Other groups at increased risk for developping severe malaria are malnourished children, elderly and those with comorbidities.^79^–^81^

## Hyper-Reactive Malaria Splenomegaly

In areas with intense transmission, repeated plasmodial infections may generate a long-term stimulation of the immune system and determine a syndrome named hyperreactive malaria splenomegaly (HMS). From the clinical point of view, most subjects present with a huge spleen (to the level of umbilicus or below), abdominal distension, and a dragging left-sided abdominal pain, sometimes so sharp to suggest perisplenitis or splenic infarction.^8^ Most of HMS subjects have also non-tender hepatomegaly. Secondary hypersplenism may be associated to HMS, showing its typical blood picture with normochromic, normocytic anaemia, reticulocytosis, leucopenia and thrombocytopenia. Polyclonal hypergammaglobulinemia with high serum concentrations of IgM, high titers of antimalarial antibodies and autoantibodies (antinuclear factor, rheumatoid factors etc) are usually present. Microscopic examination of blood films is usually negative. In industrialized countries, PCR analysis for malarial DNA can provide further evidence for diagnosis of HMS.^82^ Diagnostic criteria include: long-term stay in endemic areas, massive and persistent splenomegaly, anaemia, high levels of serum IgM, increased levels of malarial antibodies and clinical and immunological response to antimalarial treatment.^83^ Left untreated, HMS may lead to a cachectic condition with increased susceptibility to potentially fatal infections. In endemic areas, HMS can be successfully treated with antimalarial required for the duration of malaria exposure.^7^ HMS has been described in immigrants from Africa and in some European expatriates living in endemic countries.^82^,^84^–^87^ In these studies, subjects with HMS were successfully treated by a short-term antimalarial therapy.

## Conclusions

In conclusion, although fever represents the cardinal feature, clinical findings in malaria are extremely diverse and may range in severity from mild headache to serious complications leading to death, particularly in falciparum malaria. Prompt diagnosis and appropriate treatment are then crucial to prevent morbidity and fatal outcomes. In fact, severe malaria is a life threatening but treatable disease. Physicians should be aware that malaria is not a clinical diagnosis but must be diagnosed, or excluded, by performing microscopic examination of blood films.^7^ A positive blood test can quickly confirm the clinical suspicion of malaria. However, subjects with symptoms compatible with severe disease, should be treated presumptively for *P. falciparum*, even if the initial blood film is negative or microscopy, or rapid diagnostic tests, are not available.

## Figures and Tables

**Table 1:** Clinical and laboratory features of severe malaria^22^

***Clinical features:***
- impaired consciousness or unrousable coma
- prostration, i.e. generalized weakness so that the patient is unable walk or sit up without assistance
- failure to feed
- multiple convulsions (more than two episodes in 24 h)
- deep breathing, respiratory distress (acidotic breathing)
- circulatory collapse or shock, systolic blood pressure < 70 mmHg in adults and < 50 mmHg in children
- clinical jaundice plus evidence of other vital organ dysfunction
- haemoglobinuria
- abnormal spontaneous bleeding
- pulmonary oedema (radiological)
***Laboratory findings:***
- hypoglycaemia (blood glucose < 2.2 mmol/l or < 40 mg/dl)
- metabolic acidosis (plasma bicarbonate < 15 mmol/l)
- severe normocytic anaemia (Hb < 5 g/dl, packed cell volume < 15%)
- haemoglobinuria
- hyperparasitaemia (> 2% or 100,000/μl in low intensity transmission areas or > 5% or 250,000/μl in areas of high stable malaria transmission intensity)
- hyperlactataemia (lactate > 5 mmol/l)
- renal impairment (serum creatinine > 265 μmol/l)

## References

[1] Harinasuta T, Bunnang D, Wernsdorfer WH, McGregor I (1988). The clinical features of malaria. Malaria: principles and practice of malariology.

[2] Gilles HM, Warrell DA, Gilles HM (1993). The malaria parasites. Essential malariology.

[3] Krajden S, Panisko DM, Tobe B, Yang J, Keystone JS (1991). Prolonged infection with Plasmodium falciparum in a semi-immune patient. Trans R Soc Trop Med Hyg.

[4] Greenwood T, Vikerfors T, Sjoberg M, Skeppner G, Farnert A (2008). Febrile Plasmodium falciparum malaria 4 years after exposure in a man with sickle cell disease. Clin Infect Dis.

[5] Szmitko PE, Kohn ML, Simor AE (2009). Plasmodium falciparum malaria occurring 8 years after leaving an endemic area. Diagn Microbiol Infect Dis.

[6] D'Ortenzio E, Godineau N, Fontanet A, Houze S, Bouchaud O, Matheron S (2008). Prolonged Plasmodium falciparum infection in immigrants, Paris. Emerg Infect Dis.

[7] White NJ, Cook GC, Manson P, Zumla A (2009). Malaria. Manson's tropical diseases.

[8] Warrell DA, Gilles HM, Warrell DA (1993). Clinical features of malaria. Bruce-Chwatt's Essential Malariology.

[9] Price RN, Tjitra E, Guerra CA, Yeung S, White NJ, Anstey NM (2007). Vivax malaria: neglected and not benign. Am J Trop Med Hyg.

[10] Anstey NM, Russell B, Yeo TW, Price RN (2009). The pathophysiology of vivax malaria. Trends Parasitol.

[11] Rodriguez-Morales AJ, Benitez JA, Arria M (2008). Malaria mortality in Venezuela: focus on deaths due to Plasmodium vivax in children. J Trop Pediatr.

[12] Alexandre MA, Ferreira CO, Siqueira AM, Magalhaes BL, Mourao MP, Lacerda MV (2010). Severe Plasmodium vivax malaria, Brazilian Amazon. Emerg Infect Dis.

[13] Taylor TE, Strickland GT, Hunter GW, Strickland GT (2000). Malaria. Hunter's tropical medicine and emerging infectious diseases.

[14] Uzzan B, Izri A, Durand R, Deniau M, Bouchaud O, Perret GY (2006). Serum procalcitonin in uncomplicated falciparum malaria: a preliminary study. Travel Med Infect Dis.

[15] Ahiboh H, Oga AS, Yapi HF, Kouakou G, Boua KD, Edjeme N (2008). [Anaemia, iron index status and acute phase proteins in malaria (Abidjan, Cote d'Ivoire)]. Bull Soc Pathol Exot.

[16] Chiwakata CB, Manegold C, Bonicke L, Waase I, Julch C, Dietrich M (2001). Procalcitonin as a parameter of disease severity and risk of mortality in patients with Plasmodium falciparum malaria. J Infect Dis.

[17] Singh B, Kim Sung L, Matusop A, Radhakrishnan A, Shamsul SS, Cox-Singh J (2004). A large focus of naturally acquired Plasmodium knowlesi infections in human beings. Lancet.

[18] Garnham PCC (1966). Malaria parasites and other haemosporidia.

[19] Daneshvar C, Davis TM, Cox-Singh J, Rafa'ee MZ, Zakaria SK, Divis PC (2009). Clinical and laboratory features of human Plasmodium knowlesi infection. Clin Infect Dis.

[20] Baird JK (2009). Malaria zoonoses. Travel Med Infect Dis.

[21] Lee KS, Cox-Singh J, Singh B (2009). Morphological features and differential counts of Plasmodium knowlesi parasites in naturally acquired human infections. Malar J.

[22] World Health Organization (2010). Guidelines for the treatment of malaria.

[23] Warrell DA, Looareesuwan S, Warrell MJ, Kasemsarn P, Intaraprasert R, Bunnag D (1982). Dexamethasone proves deleterious in cerebral malaria. A double-blind trial in 100 comatose patients. N Engl J Med.

[24] Lewallen S, Harding SP, Ajewole J, Schulenburg WE, Molyneux ME, Marsh K (1999). A review of the spectrum of clinical ocular fundus findings in P. falciparum malaria in African children with a proposed classification and grading system. Trans R Soc Trop Med Hyg.

[25] Lewallen S, Bakker H, Taylor TE, Wills BA, Courtright P, Molyneux ME (1996). Retinal findings predictive of outcome in cerebral malaria. Trans R Soc Trop Med Hyg.

[26] Lewallen S, White VA, Whitten RO, Gardiner J, Hoar B, Lindley J (2000). Clinical-histopathological correlation of the abnormal retinal vessels in cerebral malaria. Arch Ophthalmol.

[27] Beare NA, Southern C, Chalira C, Taylor TE, Molyneux ME, Harding SP (2004). Prognostic significance and course of retinopathy in children with severe malaria. Arch Ophthalmol.

[28] Holding PA, Snow RW (2001). Impact of Plasmodium falciparum malaria on performance and learning: review of the evidence. Am J Trop Med Hyg.

[29] Omanga U, Ntihinyurwa M, Shako D, Mashako M (1983). [Hemiplegia in pernicious attacks of Plasmodium falciparum in children]. Ann Pediatr (Paris).

[30] Collomb H, Rey M, Dumas M, Nouhouayi A, Petit M (1967). [Hemiplegia associated with acute malaria]. Bull Soc Med Afr Noire Lang Fr.

[31] Brewster DR, Kwiatkowski D, White NJ (1990). Neurological sequelae of cerebral malaria in children. Lancet.

[32] Holding PA, Stevenson J, Peshu N, Marsh K (1999). Cognitive sequelae of severe malaria with impaired consciousness. Trans R Soc Trop Med Hyg.

[33] van Hensbroek MB, Palmer A, Jaffar S, Schneider G, Kwiatkowski D (1997). Residual neurologic sequelae after childhood cerebral malaria. J Pediatr.

[34] Idro R, Carter JA, Fegan G, Neville BG, Newton CR (2006). Risk factors for persisting neurological and cognitive impairments following cerebral malaria. Arch Dis Child.

[35] Carter JA, Lees JA, Gona JK, Murira G, Rimba K, Neville BG (2006). Severe falciparum malaria and acquired childhood language disorder. Dev Med Child Neurol.

[36] Carter JA, Neville BG, White S, Ross AJ, Otieno G, Mturi N (2004). Increased prevalence of epilepsy associated with severe falciparum malaria in children. Epilepsia.

[37] Calis JC, Phiri KS, Faragher EB, Brabin BJ, Bates I, Cuevas LE (2008). Severe anemia in Malawian children. N Engl J Med.

[38] Paslov G, Abdalla S, Guerrant R, Walker D, Weller P (1999). Anemia. Tropical infectious diseases: principles, pathogens, and practice.

[39] Lalloo DG, Shingadia D, Pasvol G, Chiodini PL, Whitty CJ, Beeching NJ (2007). UK malaria treatment guidelines. J Infect.

[40] Weatherall D, Wernsdorfer WH, Gregor IM (1988). The aenemia of malaria. Malaria: principles and practice of malariology.

[41] (2000). Severe falciparum malaria. World Health Organization. Communicable Diseases Cluster. Trans R Soc Trop Med Hyg.

[42] Stone WJ, Hanchett JE, Knepshield JH (1972). Acute renal insufficiency due to falciparum malaria. Review of 42 cases. Arch Intern Med.

[43] Tran TH, Day NP, Ly VC, Nguyen TH, Pham PL, Nguyen HP (1996). Blackwater fever in southern Vietnam: a prospective descriptive study of 50 cases. Clin Infect Dis.

[44] Were T, Davenport GC, Hittner JB, Ouma C, Vulule JM, Ong'echa JM (2011). Bacteremia in Kenyan children presenting with malaria. J Clin Microbiol.

[45] Mabey DC, Brown A, Greenwood BM (1987). Plasmodium falciparum malaria and Salmonella infections in Gambian children. J Infect Dis.

[46] Schwarzer E, Skorokhod OA, Barrera V, Arese P (2008). Hemozoin and the human monocyte--a brief review of their interactions. Parassitologia.

[47] Pongponratn E, Riganti M, Punpoowong B, Aikawa M (1991). Microvascular sequestration of parasitized erythrocytes in human falciparum malaria: a pathological study. Am J Trop Med Hyg.

[48] Tjitra E, Anstey NM, Sugiarto P, Warikar N, Kenangalem E, Karyana M (2008). Multidrug-resistant Plasmodium vivax associated with severe and fatal malaria: a prospective study in Papua, Indonesia. PLoS Med.

[49] Genton B, D'Acremont V, Rare L, Baea K, Reeder JC, Alpers MP (2008). Plasmodium vivax and mixed infections are associated with severe malaria in children: a prospective cohort study from Papua New Guinea. PLoS Med.

[50] Anstey NM, Handojo T, Pain MC, Kenangalem E, Tjitra E, Price RN (2007). Lung injury in vivax malaria: pathophysiological evidence for pulmonary vascular sequestration and posttreatment alveolar-capillary inflammation. J Infect Dis.

[51] Kochar DK, Saxena V, Singh N, Kochar SK, Kumar SV, Das A (2005). Plasmodium vivax malaria. Emerg Infect Dis.

[52] Kochar DK, Pakalapati D, Kochar SK, Sirohi P, Khatri MP, Kochar A (2007). An unexpected cause of fever and seizures. Lancet.

[53] Baird JK (2007). Neglect of Plasmodium vivax malaria. Trends Parasitol.

[54] Kochar DK, Das A, Kochar SK, Saxena V, Sirohi P, Garg S (2009). Severe Plasmodium vivax malaria: a report on serial cases from Bikaner in northwestern India. Am J Trop Med Hyg.

[55] Saleri N, Gulletta M, Matteelli A, Caligaris S, Tomasoni LR, Antonini B (2006). Acute respiratory distress syndrome in Plasmodium vivax malaria in traveler returning from Venezuela. J Travel Med.

[56] Lee EY, Maguire JH (1999). Acute pulmonary edema complicating ovale malaria. Clin Infect Dis.

[57] Rojo-Marcos G, Cuadros-Gonzalez J, Mesa-Latorre JM, Culebras-Lopez AM, de Pablo-Sanchez R (2008). Acute respiratory distress syndrome in a case of Plasmodium ovale malaria. Am J Trop Med Hyg.

[58] World Health Organization http://data.euro.who.int/cisid.In.

[59] Romi R, Boccolini D, D'Amato S, Cenci C, Peragallo M, D'Ancona F (2010). Incidence of malaria and risk factors in Italian travelers to malaria endemic countries. Travel Med Infect Dis.

[60] Looareesuwan S, Phillips RE, White NJ, Kietinun S, Karbwang J, Rackow C (1985). Quinine and severe falciparum malaria in late pregnancy. Lancet.

[61] Nosten F, ter Kuile F, Maelankirri L, Decludt B, White NJ (1991). Malaria during pregnancy in an area of unstable endemicity. Trans R Soc Trop Med Hyg.

[62] Boel ME, Rijken MJ, Brabin BJ, Nosten F, McGready R (2012). The epidemiology of postpartum malaria: a systematic review. Malar J.

[63] Jimenez BC, Cuadros-Tito P, Ruiz-Giardin JM, Rojo-Marcos G, Cuadros-Gonzalez J, Canalejo E (2012). Imported malaria in pregnancy in Madrid. Malar J.

[64] Whitworth J, Morgan D, Quigley M, Smith A, Mayanja B, Eotu H (2000). Effect of HIV-1 and increasing immunosuppression on malaria parasitaemia and clinical episodes in adults in rural Uganda: a cohort study. Lancet.

[65] Cohen C, Karstaedt A, Frean J, Thomas J, Govender N, Prentice E (2005). Increased prevalence of severe malaria in HIV-infected adults in South Africa. Clin Infect Dis.

[66] Hewitt K, Steketee R, Mwapasa V, Whitworth J, French N (2006). Interactions between HIV and malaria in non-pregnant adults: evidence and implications. AIDS.

[67] Abu-Raddad LJ, Patnaik P, Kublin JG (2006). Dual infection with HIV and malaria fuels the spread of both diseases in sub-Saharan Africa. Science.

[68] Shah SN, Smith EE, Obonyo CO, Kain KC, Bloland PB, Slutsker L (2006). HIV immunosuppression and antimalarial efficacy: sulfadoxine-pyrimethamine for the treatment of uncomplicated malaria in HIV-infected adults in Siaya, Kenya. J Infect Dis.

[69] Laufer MK, van Oosterhout JJ, Thesing PC, Thumba F, Zijlstra EE, Graham SM (2006). Impact of HIV-associated immunosuppression on malaria infection and disease in Malawi. J Infect Dis.

[70] Menichetti F, Bindi ML, Tascini C, Urbani L, Biancofiore G, Doria R (2006). Fever, mental impairment, acute anemia, and renal failure in patient undergoing orthotopic liver transplantation: posttransplantation malaria. Liver Transpl.

[71] Shamsi T, Hashmi K, Adil S, Ahmad P, Irfan M, Raza S (2008). The stem cell transplant program in Pakistan--the first decade. Bone Marrow Transplant.

[72] Salutari P, Sica S, Chiusolo P, Micciulli G, Plaisant P, Nacci A (1996). Plasmodium vivax malaria after autologous bone marrow transplantation: an unusual complication. Bone Marrow Transplant.

[73] Barsoum RS (2004). Parasitic infections in organ transplantation. Exp Clin Transplant.

[74] Abdelkefi A, Ben Othman T, Torjman L, Ladeb S, Lakhal A, Belhadj S (2004). Plasmodium falciparum causing hemophagocytic syndrome after allogeneic blood stem cell transplantation. Hematol J.

[75] Barsoum RS (2000). Malarial acute renal failure. J Am Soc Nephrol.

[76] Serjeant GR, Cohen J, Powderly WG (2004). Infections in Sickle Cell Disease. Infectious Diseases.

[77] Chiodini PL, Hill D, Lalloo D, Lea G, Walker E, Whitty C (2007). Guidelines for malaria prevention in travellers from the United Kingdom.

[78] Bidegain F, Berry A, Alvarez M, Verhille O, Huguet F, Brousset P (2005). Acute Plasmodium falciparum malaria following splenectomy for suspected lymphoma in 2 patients. Clin Infect Dis.

[79] Berkley JA, Bejon P, Mwangi T, Gwer S, Maitland K, Williams TN (2009). HIV infection, malnutrition, and invasive bacterial infection among children with severe malaria. Clin Infect Dis.

[80] World Health Organization (2011). International travel and health.

[81] Muhlberger N, Jelinek T, Behrens RH, Gjorup I, Coulaud JP, Clerinx J (2003). Age as a risk factor for severe manifestations and fatal outcome of falciparum malaria in European patients: observations from TropNetEurop and SIMPID Surveillance Data. Clin Infect Dis.

[82] De Iaco G, Saleri N, Perandin F, Gulletta M, Ravizzola G, Manca N (2008). Hyper-reactive malarial splenomegaly in a patient with human immunodeficiency virus. Am J Trop Med Hyg.

[83] Fakunle YM (1981). Tropical splenomegaly. Part 1: Tropical Africa. Clin Haematol.

[84] Camara B, Kantambadouno JB, Martin-Blondel G, Berry A, Alvarez M, Benoit-Vical F (2009). [Hyperreactive malarial splenomegaly: three clinical cases and literature review]. Med Mal Infect.

[85] Van den Ende J, van Gompel A, van den Enden E, Taelman H, Vanham G, Vervoort T (2000). Hyperreactive malaria in expatriates returning from sub-Saharan Africa. Trop Med Int Health.

[86] Bartoloni A, Strohmeyer M, Sabatinelli G, Benucci M, Serni U, Paradisi F (1998). False positive ParaSight-F test for malaria in patients with rheumatoid factor. Trans R Soc Trop Med Hyg.

[87] De Franceschi L, Sada S, Andreoli A, Angheben A, Marocco S, Bisoffi Z (2005). Sickle cell disease and hyperreactive malarial splenomegaly (HMS) in young immigrants from Africa. Blood.

